# Multistimuli-responsive microrobots: A comprehensive review

**DOI:** 10.3389/frobt.2022.1027415

**Published:** 2022-11-07

**Authors:** Zameer Hussain Shah, Bingzhi Wu, Sambeeta Das

**Affiliations:** Department of Mechanical Engineering, University of Delaware, Newark, DE, United States

**Keywords:** microrobots, multistimuli responsive, magnetic actuation, catalytic, optical, acoustic

## Abstract

Untethered robots of the size of a few microns have attracted increasing attention for the potential to transform many aspects of manufacturing, medicine, health care, and bioengineering. Previously impenetrable environments have become available for high-resolution *in situ* and *in vivo* manipulations as the size of the untethered robots goes down to the microscale. Nevertheless, the independent navigation of several robots at the microscale is challenging as they cannot have onboard transducers, batteries, and control like other multi-agent systems, due to the size limitations. Therefore, various unconventional propulsion mechanisms have been explored to power motion at the nanoscale. Moreover, a variety of combinations of actuation methods has also been extensively studied to tackle different issues. In this survey, we present a thorough review of the recent developments of various dedicated ways to actuate and control multistimuli-enabled microrobots. We have also discussed existing challenges and evolving concepts associated with each technique.

## 1 Introduction

Untethered, controllable, mobile microrobots with the ability to navigate into spaces at the microscale and below have been proposed for numerous applications, especially in manufacturing, biology, and medicine. The last 2 decades have seen tremendous effort in microrobotic development because of their potential to radically improve the efficacy of numerous tasks, such as targeted drug delivery, cleaning clogged arteries, cell sorting, biopsy (Jin et al., 2020), cell manipulation (Kim et al., 2013; Steager et al., 2013; Villa et al., 2018a; Hu et al., 2019), microsurgery (Vyskočil et al., 2020), and mixing of particles (Yigit et al., 2019). In addition, due to their small size, microrobots are uniquely suited for manipulating microscale parts to realize low-cost micro-assembly operations (Takahashi et al., 2016; Foroutan, 2018). The accurate and precise motion control of microrobots also makes them suitable for environmental monitoring and remediation applications (Mushtaq et al., 2015).

Although there is no concrete definition for microrobots in the literature, over the years, researchers have used the following criterion to define a microrobot (Diller and Sitti, 2013): 1) the size of all the features and the overall footprint of the robot is in the range of microns, and 2) surface related forces such as surface tension, drag, viscous forces, Brownian motion, etc. (Das et al., 2015) dominate motion of the robot, and the inertial forces become negligible because microrobots have a larger surface area compared with their small size. Because of size restrictions and special interaction forces, it is challenging to power and control multiple micron-scale robots, since the conventional components used for multiple macro-scale robots such as onboard actuators, transducers, power components, computational components (CPU) cannot be scaled down for a team of micron-scale robots. Thus, researchers have proposed non-conventional methods to power and control multiple microrobots. In terms of actuation, some of them employ off-board global fields (Kummer et al., 2010; Jing et al., 2013; Ongaro et al., 2019; Chah et al., 2020), while others rely on dedicated sources of power and actuation (Banerjee et al., 2014; Cappelleri et al., 2014). Some of the common external actuation techniques include actuation through electrophoresis employing electric fields (Kim and Kim, 2016; Kim et al., 2020), optical actuation (Jesper Glückstad, 2017), magnetic field actuation (Chowdhury et al., 2016), thermal actuation (Erdem et al., 2010; Go et al., 2018), and by attachment to the swimming bacteria (Ng et al., 2018; Chen et al., 2019a). Besides these, microrobots powered by chemical reactions have also been studied extensively. They were first reported by Whiteside’s group (Ismagilov et al., 2002), which triggered a chain reaction of discovery in this vastly unknown territory.

Microrobots can achieve propulsion in different ways. Generally, the microrobotic propulsion can be either chemically powered or an external field powered (Wang, 2013). The chemically powered micromotors convert the chemical energy from their soundings into mechanical energy (Shah et al., 2020) to drive their motion. The design of these microrobots requires an asymmetric catalytic center that can break an energy rich chemical typically hydrogen peroxide (H_2_O_2_) (Chen et al., 2019b). Since the chemical fuels are generally toxic, chemically powered microrobots offer limited biomedical applications. In recent years, there has been a significant interest to develop microrobots that utilize biocompatible fuels. The focus has been on two approaches so far to achieve this goal i.e., either employ enzymes as catalysts to drive microrobots (Ma et al., 2016; Mathesh et al., 2020; Valles et al., 2022; Villa et al., 2022), or synthesize microrobots that require biocompatible fuels such as glucose (Kwon et al., 2021), citric acid (Teo et al., 2016), and gastric acid (Zhou et al., 2019). An alternative approach is the employment of fuel-free microrobots that are actuated by external fields such as magnetic, electric, or acoustic fields. Generally, chemically powered microrobots are considered suitable for the environmental applications while the fuel-free microrobots are more attractive for the biomedical applications.

For many practical applications, microrobots actuated by a single power source face several limitations. For instance, a precise control of direction and speed of microrobots is a prerequisite in biomedical applications. Similarly, many future applications of microrobots require a collective effort by an army of these tiny machines. Such control cannot be achieved using a single actuation method. There are several other shortcomings that are associated with a particular actuation technique such as low propulsive thrust, limited workspace, etc. To overcome these issues, researchers have focused on combining multiple actuation methods. The coupling of different stimuli offers advantages that are beyond the reach of a single stimulus. For example, chemically powered microrobots are propelled spontaneously in random directions upon their contact with a fuel solution. This lack of directionality hinders their use in different applications. However, a simple addition of a magnetic component to these microswimmers imparts a precise control over the path followed by them. Similarly, an acoustic activation of chemically propelled micromotors offers an easy mechanism for the collective dynamics of these particles. Therefore, multistimuli-responsive microrobots are very promising candidates for practical applications.

One of the earliest examples of multistimuli-responsive microrobots was demonstrated by Wang’s team (Gao et al., 2011). They combined the catalytic properties of platinum and the magnetic nature of nickel to design a nanowire shaped microrobot consisting of Pt-Au-Ag-Ni segments. The team showed an excellent control over speed and direction of motion of these motors by changing the fuel concentration and applying a magnetic field, respectively. In the follow-up work, the same group reported the first fuel-free synthetic microrobot actuated by a combination of a magnetic and acoustic field (Li et al., 2015). The combination of these two stimuli enabled the microrobots to navigate in complex media such as sea water and blood. These microrobots also showed collective behavior such as aggregation and swarming. Beside these combinations, natural swimmers such as bacteria (Bastos-Arrieta et al., 2018) and sperm cells (Singh et al., 2020b) have also been coupled with the synthetic micromotors to develop “biohybrid microrobots” (Iberite et al., 2022). The biohybrid microrobots offer a facile approach to harness nature’s unique attributes such as self-healing and adaptability (Mestre et al., 2019).

**FIGURE 1:**
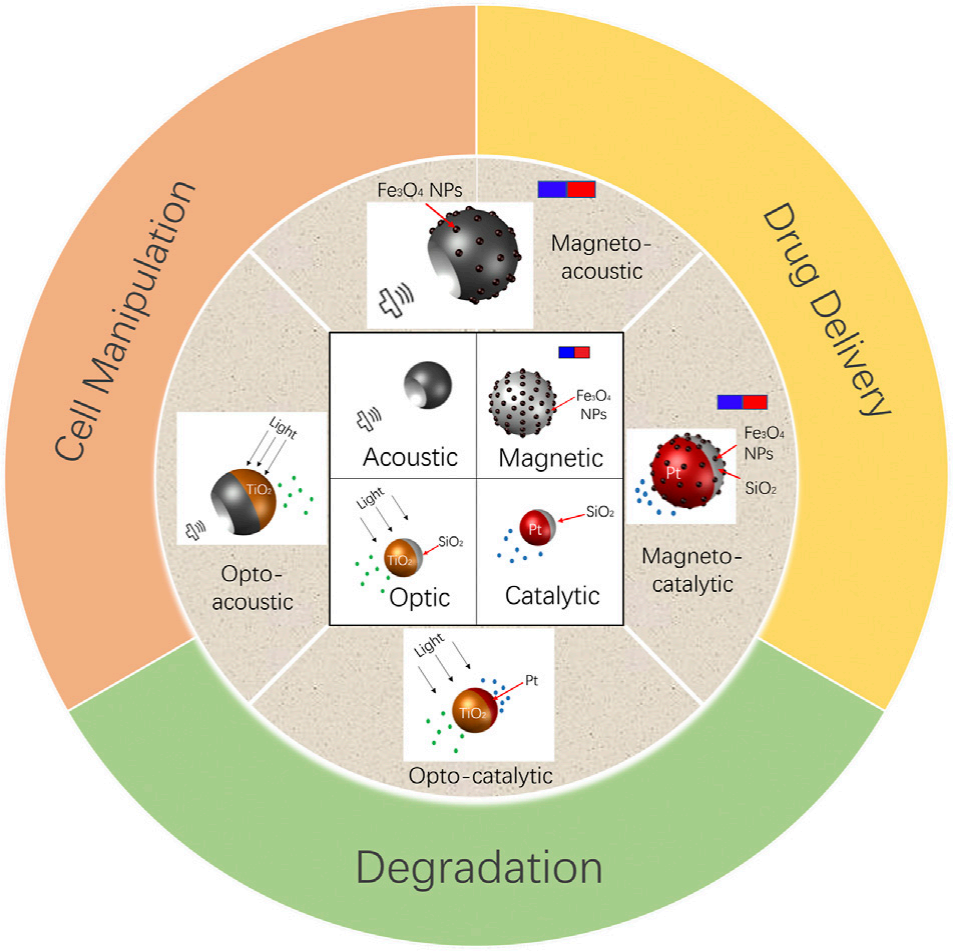
Representative examples of multistimuli-responsive microbots for different applications.

In this review, we present an overview of coupling of different actuation techniques used for the microrobotic activation. The review is divided into different sections. Each section starts with a brief description of a particular propulsion mechanism such as magnetic or acoustic actuation. This is followed by examples from literature reporting coupling of different actuation modes e.g., magneto-catalytic, etc. Then each section ends with a discussion on different applications targeted by a combination of these stimuli reported in recent years. The structure of this review is also depicted schematically in Figure 1.

## 2 Multistimuli-responsive microrobots

### 2.1 Magnetically actuated multistimuli-responsive microrobots

One of the actuation methods that gained attraction in recent years is magnetic actuation because of the numerous factors that make it well suited to actuate and control multiple microrobots. First, magnetic fields can be employed in opaque and enclosed environments and at large penetration depths. This is unlike some other actuation mechanisms, like light for example, which requires a transparent medium. Secondly, magnetic fields are considered a safe choice to use at the cellular and tissue level for many biomedical applications (Sitti et al., 2015; Sitti and Wiersma, 2020). Moreover, high actuation force, compact system size, and low hardware cost also makes it a highly popular actuation technique for microrobots. Magnetically responsive microrobots are typically made of ferromagnetic or paramagnetic material.

Magnetic actuation causes the propulsion of magnetically responsive microrobots with magnetic torques and/or forces. A low-frequency and quasi-static magnetic field is the commonly used approach to apply forces and torques directly to untethered magnetic microrobots (Nelson et al., 2010; Zhang et al., 2017b). When a microrobot having an embedded magnetic component is placed in a magnetic field, B, it experiences a magnetic force, F. The magnetic force can be calculated using the following equation (Fountain et al., 2010):

F=(m.∇)B

Where, m is the magnetic dipole moment and ∇ is the gradient of the magnetic field.

When the gradient of B is zero, the microrobot will not experience a magnetic force. The microrobots in a magnetic field can also experience the magnetic torque, τ, which is given by

τ=m×B

**FIGURE 2:**
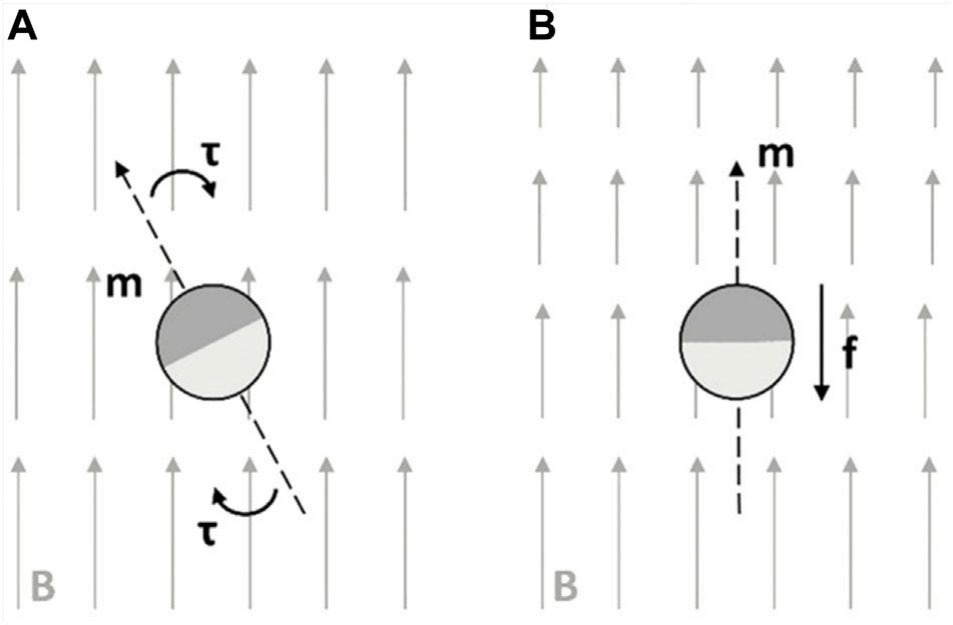
Mechanisms of magnetic propulsion **(A)** torque-dependent actuation under time-varying magnetic fields and **(B)** force-dependent actuation under non-uniform magnetic fields. Reproduced with permission (Yang and Zhang, 2020). Copyright 2020, Wiley-VCH.

The torque will force the magnetic robot to align its dipole moment with the direction of B (Koleoso et al., 2020). Therefore, the magnetic propulsion can either be achieved by time-varying magnetic fields (torque actuated) or inhomogeneous magnetic fields (force actuated). These two mechanisms are illustrated in Figure 2. Since the focus of this review is the multistimuli-responsive microrobots, we will base our discussion on the magnetically actuated hybrid microrobots in the following sections.

#### 2.1.1 Magneto-catalytic actuation

Chemical propulsion of microrobots has also been explored extensively in recent years (Zhang and Hess, 2021) due to its advantages, including ease of operation and relatively higher thrust force compared to other actuation techniques (Li et al., 2016). The chemically-powered micromotors have been inspired by the bacterial flagella that use chemical gradients to navigate (Soto et al., 2020). The basic principle of chemical propulsion is the generation of thrust force due to the reaction between two chemical species (Peng et al., 2022). A microrobot of this kind needs continuous chemical energy conversion and asymmetric shape to sustain the propulsion because of the negligible inertial forces at very low Reynolds number (Wang, 2013; Wang et al., 2013). It usually consists of an inert material to build the asymmetric shape and catalyst/active metal to trigger the chemical reaction (Lyu et al., 2021).

**FIGURE 3:**
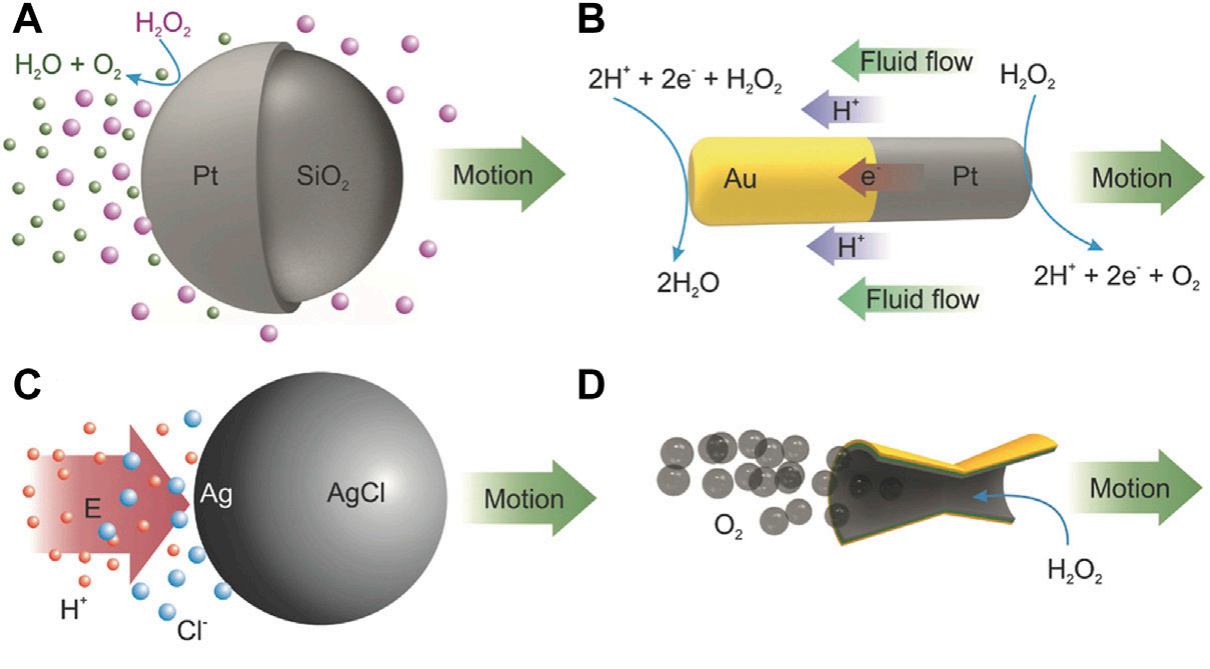
Chemical propulsion mechanisms **(A,B)** diffusiophoretic, **(C)** electrophoretic, and **(D)** bubble induced propulsion. Reproduced with permission (Safdar et al., 2018). Copyright (2018) Wiley-VCH.

The catalytic propulsion can commonly be achieved through two different means: self-phoretic propulsion and bubble-induced propulsion. The phoretic propulsion can further be divided into diffusiophoresis and electrophoresis (Safdar et al., 2018). When a Janus particle reacts with a chemical, the reaction products are generated at the catalytic surface (Figure 3A). The reaction products can be neutral or charged species (Figures 3B,C). A concentration gradient of the reaction products is developed on one side of the Janus colloid. The particle experiences more force on one side which triggers its propulsion. This phenomenon is known as diffusiophoretic propulsion. In the case of ionic reaction products, a local electric field is generated due to asymmetric formation of charged species. The propulsion in this case is called electrophoretic propulsion and it is commonly observed for the bimetallic (Au-Pt) motors (Figure 3C). Bubble propulsion has also been actively investigated, which utilizes the force produced when bubbles are continuously generated in a confined cavity through a chemical reaction and eventually emitted from the opening of the cavity (Figure 3D). The resultant recoil force propels the microrobot, which is generally fabricated in the form of a tube containing the catalytic material on its inner surface.

Catalytic propulsion is not a feasible technique for controlling multiple structurally homogeneous microrobots and requires heterogeneity in their structure for independent actuation. For the case of structurally homogeneous catalytic microrobots, there is no net gradient around particles owing to uniformly distributed reaction products, hence there is no active motion. To overcome this issue, the chemical actuation can be combined with magnetic actuation where the thrust force to move the particle is provided by the chemical actuation while the torque to orient each particle independently is provided by the magnetic actuation and hence the microrobots can be controlled independently (Villa et al., 2018a; Das et al., 2018).

As described in early reports, hybrid micromotors are a combination of any of the actuation methods discussed above. The dual action of micromotors is of particular interest for environmental and biomedical applications. For environmental remediation, a combined action of magnetic and catalytic actuation has been a great success. Polluted water has been a major concern for healthy life ever since the industrial revolution. A massive effort is being put on during the past 4 decades to get rid of industrial pollutants. Among various pollutants, nitroaromatics are of particular concern due to their abundant usage and nonbiodegradable nature (Kovacic and Somanathan, 2014). In an effort to eradicate nitroaromatics from water, Srivastava and co-workers (Srivastava et al., 2016) employed microrobots as wastewater cleaners. Specifically, they fabricated rolled-up tubular microjets made of Ti/Fe/Cr sheets with Pd particles grown onto them. They selected 4-nitrophenol as a model nitroaromatic candidate and studied its degradation by the micromotors. The catalytic degradation of 4-nitrophenol took place inside tubular motor and resulted in the release of bubbles, hence creating propulsion. This unique strategy therefore utilized pollutants as the micromotor fuel. The microjets were collected magnetically after the reaction which makes them reusable. In a similar attempt at environmental remediation, researchers employed micromotors based on the photocatalytic metal-organic frameworks for degradation of organic contaminants (Chen et al., 2020). They decorated microfluidic emulsion microdroplets with Fe_3_O_4_@Ag nanoparticles and zeolitic imidazolate framework-8@ZnO nanoparticles (ZIF-8@ZnO NPs). The Fe_3_O_4_@Ag served as an engine for the catalytic decomposition of hydrogen peroxide and MOFs offered greater surface area for the adsorption of Rhodamine dye. The enhanced degradation was achieved by a combination of UV triggered photocatalysis over ZIF-8@ZnO NPs and advanced oxidation by H_2_O_2_. The micromotors were collected magnetically for recycling. Recently, Yang and colleagues (Yang et al., 2021) have also reported a magnetic Janus micromotor for pollutants removal. They used γ-Fe_2_O_3_ particles as cores and grew amine-functionalized silica shells on them. The particles were half covered with Ag that catalyzed H_2_O_2_ decomposition to trigger the bubble-propelled motion. These micromotors showed an impressive speed of 203.06 ± 10.6 μm/*s* in a 10% H_2_O_2_ solution. These micromotors were tested for the removal of Cu^+^ and doxycycline and they were found to be three times more efficient than their non-motile counterparts. Ren and co-workers (Ren et al., 2019b) proposed bubble-propelled microrobots developed by a microfluidic method with controllable shapes and sizes for effective wastewater treatment. Fe_3_O_4_ and MnO_2_ nanoparticles were successfully loaded on Janus micromotors. Fe_3_O_4_ nanoparticles controlled the movement direction of microrobots and acted as catalysts for pollutant degradation. MnO_2_ nanoparticles on the concave of the microrobots catalyzed H_2_O_2_ to produce bubble-propelled motion in solution. Another intriguing example of the bubble-propelled micromotors for environmental remediation is the successful demonstration of radioactive waste i.e. uranium removal from an aqueous solution (Ying et al., 2019). These micromotors were based on metal-organic frameworks incorporating magnetic Fe_3_O_4_ nanoparticles and Pt catalyst. The microrobots showed a 96% removal of uranium in the presence of 1% H_2_O_2_ which was much higher than (13%) that achieved by same particles in static state.

Besides organic pollutants, oil spills and plastic wastes are also a major cause of water pollution. To tackle this issue, researchers have successfully developed microrobots to get rid of the spilled oil and unwanted polymers. Mou et al. (2015b) developed an asymmetric pot-like MnFe_2_O_4_ micromotor with a single hole in it. The motor was propelled by the catalytic decomposition of H_2_O_2_ and further guided by a magnetic field. Moreover, the authors compared the motion of these micromotors when actuated by a single stimulus or two stimuli. They demonstrated a fine control over the micromotor propulsion by applying magnetic field. Specifically, they were able to stop the catalytically propelled motor when a magnetic field was applied opposite to the direction of motion. The magnetic force exerted to stop the motion was calculated to be ×6.5 10^–11^ N which was close to the micromotor driving force of 5.8 × 10^–11^ N. Similarly, the propulsion speed was increased from 200 μm/s to 390 μm/s when both the magnetic field and catalytic propulsion were in the same direction. The magnetic force applied on the micromotor in this case was ×1.17 10^–11^ N This kind of propulsion control is rarely reported and could be very useful for different applications. The micromotor was made hydrophobic during the synthesis that allowed it to collect oil from the environmental samples. Similarly, bubble-propelled micromotors synthesized by coating Pt and Ni on carbon soot were found efficient in the oil-spill recovery (Singh et al., 2020a). The authors also showed that the applied magnetic field would increase the speed of catalytically powered micromotors. They were able to determine and increase in speed from 9.8 μm/s in 10% H_2_O_2_ to 15.3 μm/s when a magnetic field of 50 mT was applied. Gao et al. (Gao et al., 2013) have also shown that a micromotor made of Mg microparticles with a Ni-Au bilayer could effectively collect the oil droplets from seawater without any fuel. For practical applications, an easy implementation of microrobots for treatment of bigger volumes and a facile collection for reusability are highly desirable. A very recent report from Sanchez’s group has addressed both of these issues (Vilela et al., 2022). The authors introduced a new concept of “micromotor-in-sponge”. They used polyurethane-based material as sponge and embedded cobalt-ferrite (CFO) micromotors into it. This coupling allowed the enhanced capturing of pollutants due to porosity of the sponges and *in-situ* degradation by the catalytic micromotors. The authors demonstrated an impressive multicycle pollutant treatment of 1 L sample in 15 min. They employed magnetic attraction for the facile collection of these hybrid microrobots for reuse.

The hybrid action of the magnetocatalytic microrobots has also been explored in biomedical applications. In this context, multifunctional microrobots made of superparamagnetic polymeric particles coated with Pt have been reported for cell manipulation and anticancer drug delivery to breast cells (Villa et al., 2018a). Similarly, researchers (Hwang et al., 2019) have developed a micromotor for biofilm eradication. The authors designed antimicrobial microrobots loaded with iron oxide NPs that offered a dual magneto-catalytic functionality. These microrobots could break the biofilm exopolysaccharide matrix by generating bactericidal free radicals. The broken fragments of the biofilm were removed by the magnetically driven assemblies of these robots in a magnetic field of 3.4 mT intensity. Moreover, the authors experimentally confirmed the synergistic effect of magneto-catalytic properties of their microrobots for the biofilm eradication. They showed that microrobots driven by magnetic field could cut through parts of biofilm, however, bacterial cells remained viable after this treatment. On contrary, the catalytically active microrobots (in 1% H_2_O_2_) driven by the same magnetic force could completely remove the biofilm causing death of all the exposed bacteria.

The combined action of magneto-catalytic microrobots has also been studied in sensing. The presence of bacterial endotoxins can result in organ failure and septic shock. Their rapid detection can be a life saving tool in certain scenarios. Jurado-Sanchez et al. (Jurado-Sánchez et al., 2017) have developed an ultrafast sensor system for the detection of bacterial endotoxins. Their sensors were based on a Janus micromotor encapsulating Phenylboronic acid (PABA) modified graphene quantum dots (GQDs). The micromotor was loaded with Pt and Fe_3_O_4_ for catalytic and magnetic activation, respectively. The researchers used 5% H_2_O_2_ for bubble propulsion and 0.57 T surface field strength for magnetic actuation of these micromotors. The sensing was based on the fluorescence quenching resulting from the interaction of GQDs with the bacterial endotoxins. Just like the toxic bacterial endotoxins, the detection of chiral enantiomers is also very important in life sciences. Chiral molecules are common in nature and their two enantiomeric forms can have completely different effects on the living systems. Recently, Pumera’s group (Muñoz et al., 2022) have developed a hybrid micromotor for the enantiorecognition of tryptophane enantiomers. Their micromotor was made of Ni@Pt microrockets loaded with CdS quantum dots that carried *ß*-cyclodextrin. As *ß*-cyclodextrin is well known for its affinity towards different chiral molecules, the researchers were able to discriminate between tryptophane enantiomers by employing their microrobots. The authors used 1% H_2_O_2_ and 3 mT magnetic field intensity as operating conditions for their microrobots.

#### 2.1.2 Magneto-acoustic actuation

Although magnetic field and light are the most commonly used actuation systems for micro scale manipulation with multiple microrobots, their output resultant force is low in magnitude (Petit et al., 2012; Brzobohatý et al., 2013; Gao et al., 2017). Moreover, they require intricate assembly and are exceedingly expensive. The holy grail of microrobotics is their biomedical applications. The scale of those applications demands an easily approachable actuation setup for the synthetic micromotors. Acoustic actuation of micromotors offers the advantages of long life, on-demand motion control, biocompatibility and contactless noninvasive procedure (Xu et al., 2017). Therefore, there has been a great surge in this area ever since Mallouk and co-workers published the first ever report on ultrasound-propelled micromotors (Wang et al., 2012). Another unique advantage of acoustics over other field-driven systems is that this approach allows independent activation and propulsion of each swimmer in a group, which can be a powerful tool in collaborative functions (Ahmed et al., 2015).

The acoustic actuation enables the accumulation of multiple micro-objects at targeted sites due to predictable motion of the micro-objects controlled by applied wave functions. Acoustic fields are used to propel the microrobots remotely in a microfluidic space and the physical effects appear in the forms of an acoustic streaming and acoustic radiation forces. When a standing acoustic wave is applied in a resonator, the wave reflects back and forth and at the same time generates an acoustic pressure gradient which provides a hydrodynamic drag force, regarded as an acoustic radiation force (F_rad_), driving the micro-objects to the acoustic pressure nodes and anti-nodes (King, 1934; Yosioka and Kawasima, 1955; Shi et al., 2009). The magnitude of F_rad_ can be calculated by the following equation (Palmeri et al., 2005):

$$F_{rad}=2\alpha I/c$$

Where, F_rad_ is the force on an object, α is the absorption coefficient of the medium, I is the temporal average intensity at that spatial location, and c is the speed of sound.

The acoustic radiation forces are classified as primary radiation forces and secondary radiation forces (Weiser et al., 1984). The interaction between the acoustic field and a particle results in the generation of primary radiation forces. While the acoustic waves reflected from a particle can produce secondary radiation forces that act on an adjacent particle. The primary radiation forces are accounted for the migration of microrobots in an acoustic field and the secondary radiation forces are responsible for the particle-particle interactions (Li et al., 2022). The acoustic actuation could induce collective dynamics of microswimmers (Mettin et al., 1999; Lazarus et al., 2017) which is more attractive for practical applications. For more details on the propulsion mechanism of acoustically-driven microswimmers, readers are encouraged to read excellent review articles on acoustic manipulation of microrobots (Rao et al., 2015; Xu et al., 2017; Athanassiadis et al., 2022; Li et al., 2022).

**FIGURE 4:**
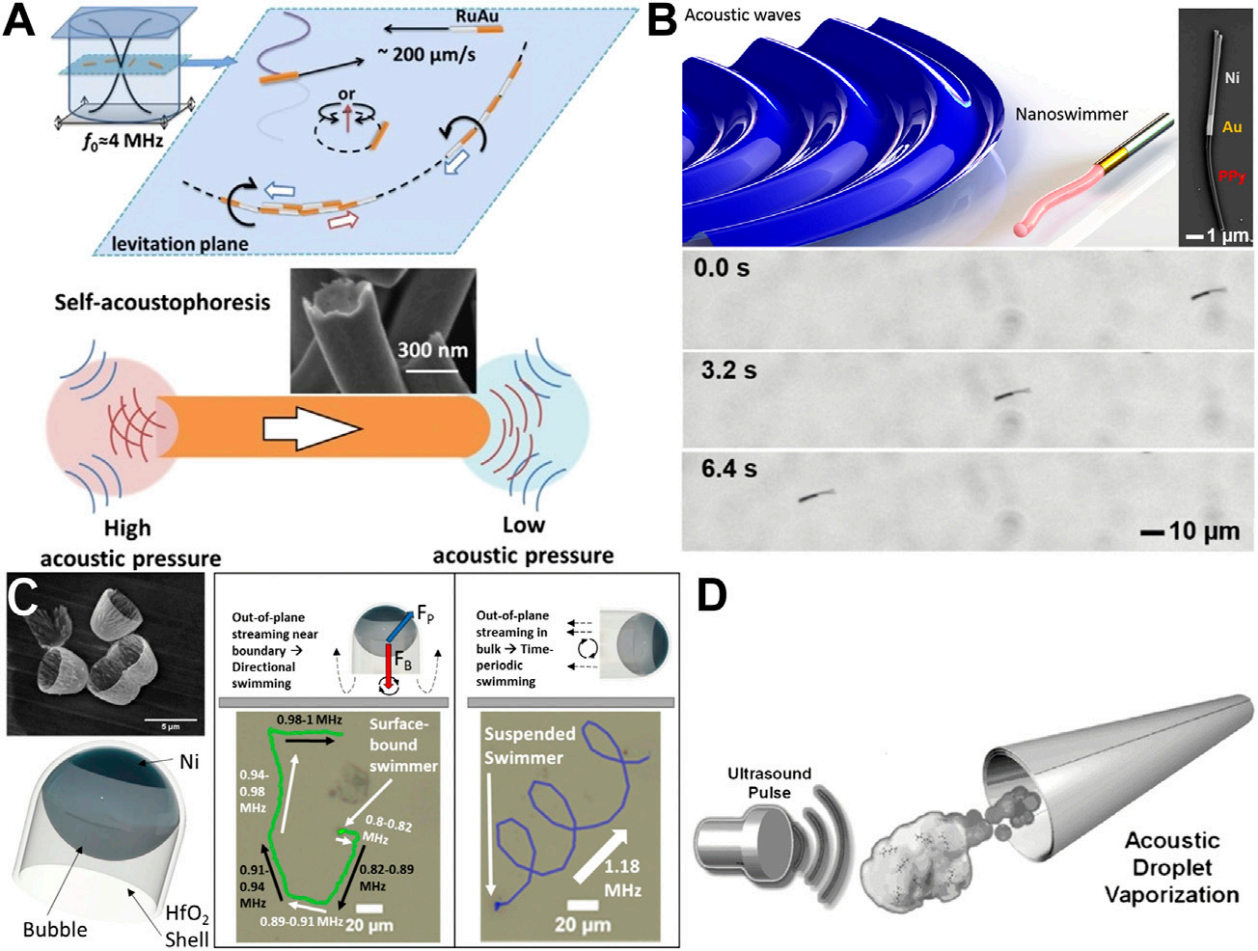
Examples of acoustically powered microrobots. **(A)** Asymmetric metallic rods propelled by self-acoustophoresis. Reprinted with permission (Wang et al., 2012). Copyright 2012 American Chemical Society. **(B)** Bimetallic rods propelled by acoustically activated flagella. Reproduced with permission (Ahmed et al., 2016a). Copyright 2016 American Chemical Society. **(C)** SEM image and schematic of cup-shaped swimmers along with different propulsion behavior at different acoustic frequencies. Reproduced with permission (McNeill et al., 2020). Copyright 2020 American Chemical Society. **(D)** Metallic microtubes propelled by acoustically vaporized bubbles of perfluorocarbon. Reproduced with permission (Kagan et al., 2012). Copyright 2012 Wiley-VCH.

The propulsion of micro/nanoparticles by ultrasound has long been observed. The first systematic attempt to explain the mechanism of acoustic propulsion was reported by Wang at al. (Wang et al., 2012). They showed that their metallic microrods could be levitated, rotated, propelled, aligned, and assembled in a 3.7 MHz acoustic field frequency in an aqueous environment or highly ionic solutions (Figure 4A). More intriguingly, the bimetallic microrods made of Au/Ru or Au/Pt always exhibited a unidirectional motion with the Ru or Pt end leading. They attributed this unidirectional propulsion to the shape asymmetry of the microrods. The authors observed that the Au end of the microrods was always of concave shape while the other end was of slightly convex shape or flat. That could lead to an asymmetric acoustic force on the rods with the Au end experiencing greater pressure. They coined the term “self-acoustophoresis” to describe this means of propulsion. A similar acoustic propulsion mechanism was reported by Joseph Wang’s group (Garcia-Gradilla et al., 2013). They fabricated a Au/Ni/Au nanowire and deliberately made one end concave which greatly enhanced the speed of the micromotor powered by an acoustic transducer at 2.51 or 2.66 MHz acoustic field frequency. Nonetheless, Nadal and Lauga (Nadal and Lauga, 2014) provided a theoretical model for the experimental observations of Wang at el. (Wang et al., 2012). and they came up with a different explanation of the actuation mechanism. Their theory is based on asymmetric steady fluid streaming that could induce the propulsion of hard particles in a standing acoustic wave. The theory was experimentally supported by Ahmed et al. (Ahmed et al., 2016b). The streaming model relies on a standing acoustic wave which is not very attractive for the *in vivo* applications. As an alternative, Ahmed et al. introduced a new class of acoustically propelled swimmers based on the oscillation of a flagellum-like flexible structure (Figure 4B) (Ahmed et al., 2016a). The swimmer is comprised of a bimetallic head and a soft tail that oscillates during the acoustic activation at −91 kHz frequency. The swimmer showed a translational motion at this frequency (Figure 4B). Moreover, the motor is propelled by a standing as well as a traveling wave. A bubble trapped in a microchannel could also cause propulsion by its oscillation activated by an acoustic force. This concept was introduced by Dijkink et al. (Dijkink et al., 2006) who demonstrated the idea of the underwater propulsion employing acoustic streaming as result of the oscillation of an acoustically excited gaseous bubble at 1.55 kHz frequency trapped in a sub-mm scale channel. Even though the proposed acoustic bubble-based propulsion method was suitable for *in-vivo* manipulation due to biocompatibility and capability of remote propulsion, it is validity in microfluidic workspace was contentious. Hence, Feng et al. (Feng and Cho, 2013; Feng et al., 2015) proposed and verified the micro propulsion *via* acoustic excitation of a bubble trapped in a micro channel through CFD simulations and experiments. They found that the bubble oscillation and hence the propulsion speed was maximum at the resonance frequency of 11.2 kHz. Later, Ahmed et al. demonstrated selective acoustic excitation of trapped microbubbles with different sizes and showed 2D navigation of an acoustic bubble-driven microrobot (Ahmed et al., 2015). They showed that the trapped bubbles of different sizes required different resonance frequencies for their optimum propulsion. Similarly, Jang et al. (Jang et al., 2018) have demonstrated the generation of sufficient force to propel microrobots and harvest acoustic energy by acoustically oscillating bubbles trapped in a microrobot. They showed that miniature rotors actuated by acoustically oscillating bubbles at a frequency of 2.15 kHz could periodically vibrate piezocantilevers to generate electric power. A more advanced study on acoustic propulsion based on bubble oscillation is reported by Ren and co-workers. (Ren et al., 2019a). Their microrobots were capable of autonomous motion in three dimensions and could selectively transport individual synthetic colloids and mammalian cells in a crowded group without surface modification, labeling, or affecting nearby objects. Their motion did not require operation at acoustic pressure nodes, enabling propulsion at low power and far from an ultrasonic transducer. They used acoustic frequencies in the range of 1–3 MHz to actuate these microrobots. For practical applications of these bubble-oscillating microrobots, a large-scale synthesis has rarely been reported. To tackle this issue, researchers from Mallouk’s group have reported a wafer-scale production of acoustically driven microrobots (McNeill et al., 2020). They employed shadow nanosphere lithography and subsequent etching to fabricate entire wafers (>10^9^) of cup-shaped swimmer (Figure 4C) made of HfO_2_ with a Ni layer. Interestingly, these microrobots showed different swimming behaviors at different acoustic frequencies. Another interesting case of acoustic propulsion is the bubble generation through acoustic vaporization reported by Kagan and co-workers (Figure 4D) (Kagan et al., 2012). They employed ultrasound to vaporize the perfluorocarbon (PFC) emulsions embedded inside the rocket-shaped microrobot. Upon acoustic activation, the vaporized PFC expelled out from the micromotor and caused it to move forward. The micromotor was used for deep tissue penetration and deformation.

The integration of the magnetic field steering with the acoustic field enables more control over the motion of microrobot. Both the magnetic and acoustic actuations can be used as an independent stimulus for propulsion. However, mostly acoustic forces are employed for actuation and the magnetic component is primarily used to control the direction of motion (Mohanty et al., 2020). The magneto-acoustic combination offers a fuel-free propulsion which makes it very attractive for the biomedical applications. It also lowers the force output and offers an on/off actuation. In an early effort to combine magnetic and acoustic forces in active particles, Ahmed et al. presented the *in-vitro* propulsion of Au–Ru bimetallic nanowire-based microrobots by combining magnetic and acoustic actuation in a biocompatible medium (Ahmed et al., 2013). They demonstrated the feasibility of these microrobots for the biomedical applications by maneuvering nanomotors toward live HeLa cells in an aqueous phosphate buffer by using the multi-actuation technique. Ahmed et al. (Ahmed et al., 2017) developed a novel propulsive mechanism based on combined ultrasound and magnetic actuation modalities inspired by neutrophils rolling on endovascular walls before transmigrating to the disease site. Their magnetic particles aggregated into a rolling sphere due to the dipole-dipole interaction in the presence of a rotating magnetic field. The aggregate then migrated toward the wall due to the radiation force of an acoustic field. By combining magnetic and acoustic fields, a rolling-type motion along the boundaries was achieved. A recent study from Misra’s laboratory (Mohanty et al., 2022) reported a cephalopod-inspired microrobot powered by magneto-acoustic forces. These microrobots could trap an array of microbubbles thus mimicking the pumping mechanism of the Cephalopoda family. The better design of these microrobots offered an enhanced lifetime since their propulsion was based on multiple bubbles. The authors also studied the potential use of these microrobots in clinical applications such as localized payload manipulation and detection with ultrasound imaging.

Microrobots offer an attractive approach for the targeted delivery of therapeutics in cancer treatment (Schmidt et al., 2020). Acoustic microrobots are of significant potential towards this goal. Uygun and co-workers demonstrated that acoustically-propelled nanowires made of Au/Ni/Au/PEDOT-polypyrrole-COOH segments loaded with asparaginase enzyme could be used in effective inhibition of El4 lymphoma cancer cells (Uygun et al., 2017). The synthetic microswimmers are considered as intruders by the immune system of living organisms and therefore can suffer heavy losses once they are in the bloodstream. A better approach for biological applications is the use of biohybrid micromotors. In this context, researchers have developed an acoustically-propelled micromotor made of red blood cells (Wu et al., 2014). The RBC motor was magnetized by asymmetric distribution of iron oxide nanoparticles. Because of excellent biocompatibility, the motor showed efficient propulsion in undiluted blood and other biological fluids.

#### 2.1.3 Magneto-optical

**FIGURE 5:**
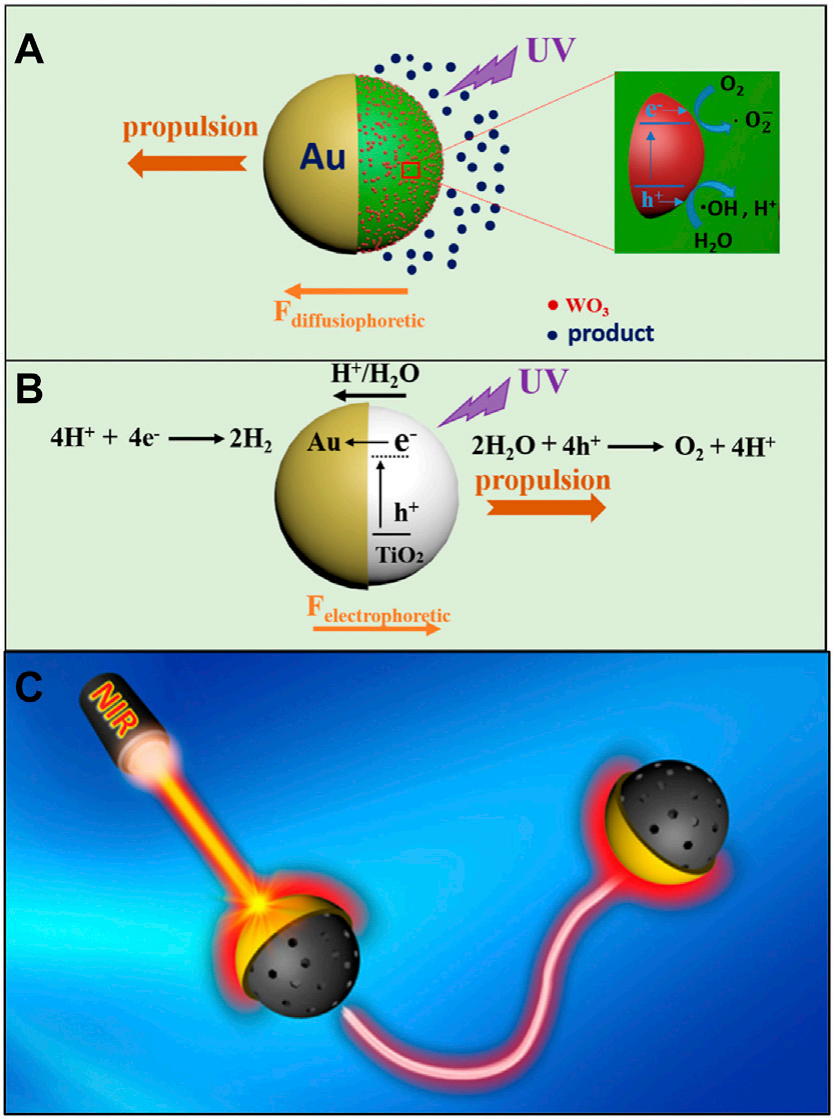
Mechanisms of light-actuated propulsion. **(A)** Self-diffusiophoresis, **(B)** Self-electrophoresis. Reproduced with permission (Zhang et al., 2017c). Copyright 2017 American Chemical Society. **(C)** Self-thermophoresis. Reproduced with permission (Xuan et al., 2016). Copyright 2016 American Chemical Society.

Light actuation of micromachines can be achieved by several mechanisms. Most of the light-powered micromotors require a Janus geometry for the asymmetric chemical reactions (Šípová-Jungová et al., 2020). A Janus micromotor is typically fabricated by deposition of a metallic catalyst on one side of a polymer or a non-active colloid (Xiao et al., 2020). Among various Janus geometries, spherical Janus colloids offer more advantages mainly in their ease of fabrication and relatively high speeds even at very low light intensities (Katuri et al., 2017). In a typical light-activated Janus micromotor, propulsion is initiated by shining light on the surface of a micromotor. Depending on the reaction conditions, the light can mainly trigger three kind of propulsion mechanisms: self-diffusiophoresis (Chen et al., 2017; Basharat et al., 2022), self-electrophoresis (Dong et al., 2017), or self-thermophoresis (Jiang et al., 2010; Xuan et al., 2016). A concentration gradient of the products across the Janus particle leads to the self-diffusiophoretic propulsion (Figure 5A). In such a scenario, higher concentration of product species on one side of the particle create an osmotic flow that propels the particle away from the photocatalytic side of the micromotor (Zhang et al., 2017c). In the case of self-electrophoresis, the propulsion is caused by a photocatalytically generated electric field (Dong et al., 2016). This propulsion mechanism has been observed in Au-TiO_2_ micromotors (Figure 5B). Upon UV light irradiation, photoexcited electrons and holes are generated inside the TiO_2_. The electrons from the conduction band of TiO_2_ get transferred into the Au hemisphere. In a typical redox reaction, protons are produced by the oxidation of water on the surface of TiO_2_ while they get reduced at Au surface. The resulting flux of protons generates a flow which propels the micromotor with TiO_2_ side leading. A few reports have also demonstrated a light-activated bubble-propelled mechanism for the actuation of tubular micromotors. These tubular microrobots were made of TiO_2_ (Giudicatti et al., 2014; Mou et al., 2015a; Enachi et al., 2016) or metal-free C_3_N_4_ (Villa et al., 2018b). Self-thermophoresis is observed when a Janus colloid is half-coated with a photothermal material mostly Au (Xuan et al., 2018). Upon irradiation from a Near Infrared (NIR) light source (Chen et al., 2022), a temperature gradient is generated at the Au side that causes the particle to move in the opposite direction (Figure 5C). Readers are encouraged to find detailed discussion elsewhere (Villa and Pumera, 2019).

Villa and co-workers (Villa et al., 2020) have designed microrobots for preventing the microbial contamination of beer. The authors targeted the removal of yeast cells from beer samples. They fabricated a micromachine consisting of BiVO_4_ and Fe_3_O_4_. The micromotors were actuated by visible light irradiation due to the excellent photocatalytic properties of BiVO_4_. Moreover, the photocatalytically active BiVO_4_ gets attached to the yeast cell and detaches upon switching off the light (Villa et al., 2019). The authors exploited this feature of BiVO_4_ combined with the magnetic NPs to remove 100% of the yeast cells from beer samples.

Polymers and plastics are an emerging concern for the future of aquatic life. For addressing this issue, Urso et al. (Urso et al., 2021) fabricated a hematite-based micromotor that showed promising results for the photodegradation of polyethylene glycol. The authors coated different metals on the hematite microspheres and found that a bimetallic coating of Pt-Pd lead to a much-enhanced propulsion speed. The micromotors were powered by a light (365 nm wavelength and 500 mW/cm^2^ intensity) and guided by a magnetic field. These micromotors were based on metal-organic frameworks incorporating magnetic Fe_3_O_4_ nanoparticles and Pt catalyst.

#### 2.1.4 Magnetic plus others

Nature uses enzymes as catalysts to speed up its reaction rates required to sustain life at all scales (Hauer, 2020). Interestingly, enzymes could be employed to power motion at the nanoscale (Zhao et al., 2018). Just like a classical catalytic micromotor, enzymes could serve as engines when attached to the passive colloidal particles (Gáspár, 2014; Mathesh et al., 2020). Catalase is the well-known enzyme for degradation of hydrogen peroxide inside the cells (Alfonso-Prieto et al., 2009). Moreover, the degradation products are water and oxygen, hence the reaction is exactly like the decomposition of H_2_O_2_ over Pt. Therefore, it is not surprising that researchers have tried to replace Pt with catalase to make an enzyme-powered micromotor. In a pioneering work, Sanchez and co-workers loaded catalase onto a rolled-up microtube (Sanchez et al., 2010). These biohybrid microengines proved to be more powerful and more efficient than those of Pt based bubble-propelled tubular micromotors. Following their success with the enzyme-propelled tubular micromotor, same group utilized catalase to power a Janus micromotor made of mesoporous silica nanoparticles half-covered with a Ni coating (Ma and Sanchez, 2015). The direction of motion of this motor was guided by an external magnetic control and the mesoporous nature of the silica was exploited for cargo loading. Apart from catalase, Urease has also been reported as a promising candidate for powering microrobots for *in vivo* applications (Patiño et al., 2018; Arqué et al., 2022). The catalytic action of urease has been combined with the magnetic activation for controlling the direction of motion in liquids of viscosities comparable to that of blood (Luo et al., 2020) and drug delivery (Ma et al., 2016).

The motion of the living organisms can be controlled by exploiting their physiological energy. The overall system comprises of naturally produced or artificially modified living organisms, a navigating system that can be either phototactic, magnetotactic, or chemotactic to control their motion, and the CCD camera attached to a microscope to obtain the visual feedback. Kim and co-workers (Kim et al., 2015) made a system by using the motility of a eukaryotic cell *Tetrahymena* pyriformis. Pyriformis cells are made responsive to the magnetic field by treating them with ferromagnetic nanoparticles. Hence, the motion of the Pyriformis cells can be controlled by binary switching of the electromagnetic coils while the cells move randomly in the absence of the magnetic field.

Becker et al. (Becker et al., 2013; Becker et al., 2014) used the global magnetic field to independently control multiple T. pyriformis cells by exploiting the non-homogeneity of the magnetic material consumption by each cell. The time for cell orientation towards the magnetic field direction was directly proportional to the amount of magnetic material consumed by the cell. The authors developed a feedback controller to individually control multiple cells by using heterogeneity among the cells, but the cells failed to demonstrate complex motion such as obstacle avoidance and shortest path owing to the single global input.

A new class of microrobots created by coupling sperm cells to mechanical loads has shown very promising results for potential biomedical applications (Singh et al., 2020b). Spermbots utilize the flagellar movement of the sperm cells for propulsion and as such do not require any toxic fuel in their environment. They are also biocompatible and demonstrate considerable speed thereby overcoming the challenges of propulsion and biocompatibility. Magdanz et al. (Magdanz et al., 2020) demonstrated the control of multiple IRONSperms by a rotating magnetic field produced around an arbitrary axis in space generated using a triaxial Helmholtz electromagnetic coil setup.

#### 2.1.5 Multistimuli swarms: Magnetic actuation

There are many complex tasks that require a highly intelligent collective effort and cannot be performed individually (Mou et al., 2020; Kaspar et al., 2021). This is true even at the nanoscale (Zhang et al., 2017a). Therefore, for the future practical applications of microrobots, it is highly imperative to design and study the collective performance of micromachines (Wang et al., 2015a). To realize this, scientists have studied swarms of nano/micromotors. Magnetic actuation has proved to be highly effective towards achieving collective behaviors of microrobots. However, magnetic field alone offers very limited collective dynamics and a combination of magnetic actuation with other actuation strategies can result in more versatile collective states (Jin and Zhang, 2022).

**FIGURE 6:**
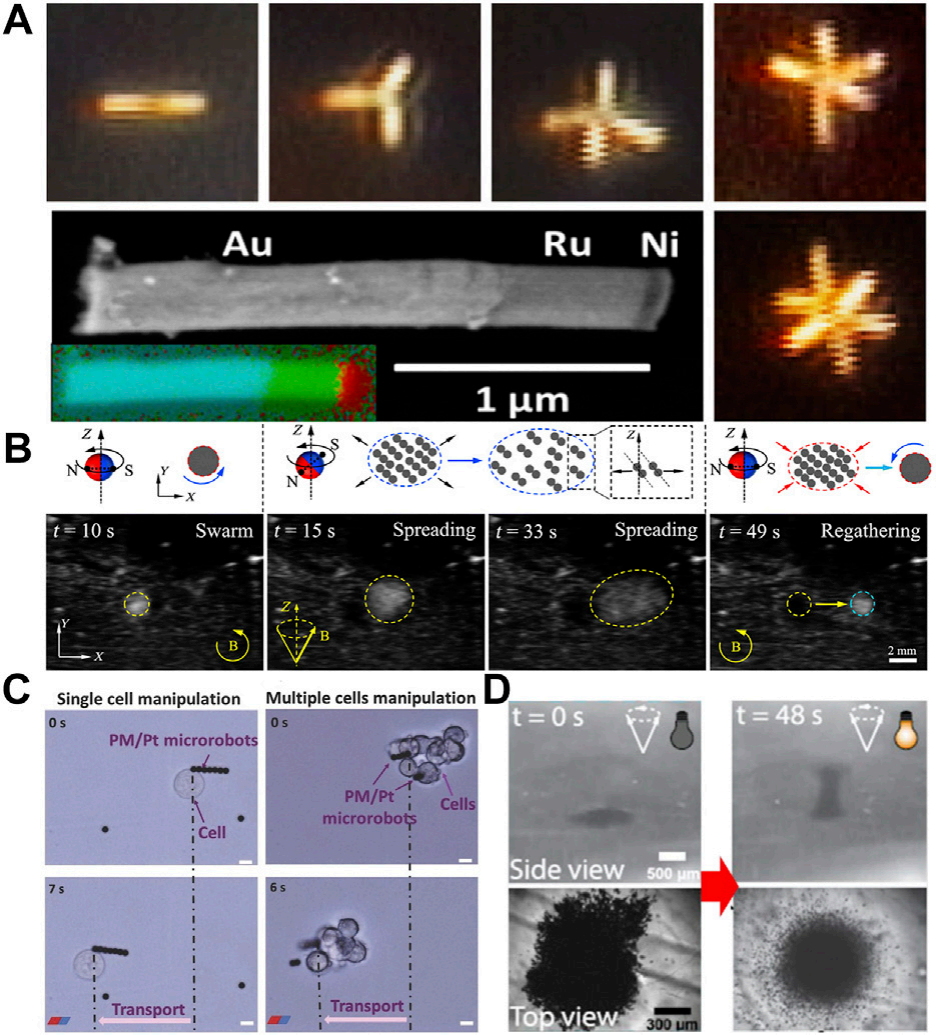
Examples of different tasks performed by magnetically guided multistimuli responsive microrobots. **(A)** Self-assembly of nanorods into geometrically regular multimers. Reprinted with permission (Ahmed et al., 2014). Copyright 2014 American Chemical Society. **(B)** Experimental results of the reversible spreading and regathering of nanoparticles in a swarm. The applied fields are shown schematically. (Wang et al., 2021). Copyright 2021 American Association for the Advancement of Science **(C)** Cell manipulation by a chain of microrobots. Reproduced with permission (Villa et al., 2018a). Copyright 2018 Wiley-VCH. **(D)** 3D tornado-like swarming pattern formation. Reproduced with permission (Ji et al., 2020). Copyright 2020 American Chemical Society.

Since most of early work on acoustic microrobots was based on the rod-shaped colloids, it was natural to add a magnetic or catalytic component to the rods to study the synergistic effect of two different actuation methods. A team of researchers lead by Mallouk (Ahmed et al., 2014) synthesized gold-ruthenium rods with a thin Ni segment to investigate the combination of magneto-acoustic fields for collective behaviors. The rods were shown to magnetically self-assemble into regular dimers, trimers, and multimers (Figure 6A). These assembled rods were levitated and propelled in fluids by applying an acoustic field of −4 MHz. The combination of magneto-acoustic fields-driven swarms are of particular importance for biomedical applications. One such application has been recently demonstrated by Zhang and co-workers (Wang et al., 2021). This team developed a magnetic microswarm guided by ultrasound for localized delivery in biological environments. They developed a strategy to navigate nanoparticle microswarms guided by ultrasound doppler imaging. Moreover, these swarms showed a reversible spreading and regathering feature depending on the applied magnetic field (Figure 6B). This combination showed promising results for active endovascular delivery. Similarly, the addition of a catalytic segment to a magnetic microrobot also offers a great combination for various applications. To realize this, researchers lead by Pumera (Villa et al., 2018a) created microrobots by coating Pt on the paramagnetic polymeric particles having a tosylated external surface. These particles were assembled into long chains by applying a weak magnetic field and then driven by the catalytic decomposition of H_2_O_2_. Interestingly, the particles could capture cells due to the tosyl group on their surface. The researchers exploited all these features of these microrobots for the manipulation of breast cancer cells (Figure 6C) in a cell culture medium using 2.5% H_2_O_2_. These microrobots also showed promising anticancer drug delivery to the cancer cells.

Most of the swarming studies have focused solely on horizontal motion in one plane. However, vertical motion of swarms is also very important especially for biomedical applications. Towards this end, Zhang and co-workers (Ji et al., 2020) introduced a light-driven tornado-like microswarm comprising of Fe_3_O_4_@SiO_2_ nanoparticles. These particles were responsive to the magnetic field while also transforming light into heat energy. The synergistic effect of magnetic field and light resulted in the formation of a vertical microswarm (Figure 6D). The applied magnetic field caused the formation of a 2D microswarm due to the hydrodynamic interactions. When a laser was illuminated on the 2D microswarm, a temperature gradient was developed around the system with the center being the hottest spot. This temperature difference produced convection flows near the microswarm in which the horizontal slip flow pushed the particles towards the center and the upward flow caused the particles to rise thus forming a tornado-like 3D structure. These microswarms were shown to be effective for increasing the degradation of methylene blue. Like the vertical propulsion, the ability to move against the flows, also known as positive rheotaxis, is also very important for therapeutics and non-invasive surgery. Recently, researchers working in Nelson’s group (Ahmed et al., 2021) reported their ground breaking findings for the positive rheotaxis achieved by microrobotic swarms. They employed magneto-acoustic fields to form and propel the microswarms against flows. The microswarms were formed by the self-assembly of superparamagnetic particles in response to a rotating field. Then these microswarms were acoustically guided towards the walls of a microchannel. These microswarms were then propelled in a rolling fashion against the direction of the fluid flow inside the microchannel.

### 2.2 Other combinations of actuation methods

#### 2.2.1 Opto-catalytic

A chemical reaction results in the propulsion of catalytic micromotors. The propulsion continues depending on the fuel availability or activity of the catalyst. Since most of the earlier work on microrobots focused on catalytic micromotors, it was natural to look for ways to control the catalytic propulsion. In this regard, researchers working in Schmidt’s group (Solovev et al., 2011) reported the control of catalytic propulsion by employing light. Their microrobots were made of rolled-up Ti/Cr/Pt microengines propelled by a bubble in an aqueous solution of H_2_O_2_ and surfactant. Interestingly, under light illumination, the microrobots stopped generating bubbles which halted their propulsion. The authors attributed this observation to a local decrease in the concentration of fuel and surfactant. The microrobots were reactivated once the light was turned off. Similarly, Duan and co-workers (Duan et al., 2013) demonstrated the control of two different collective behaviors of micromotors using opto-catalytic actuation. They could achieve either schooling or exclusion of the micromotors by using light or chemical actuation.

**FIGURE 7:**
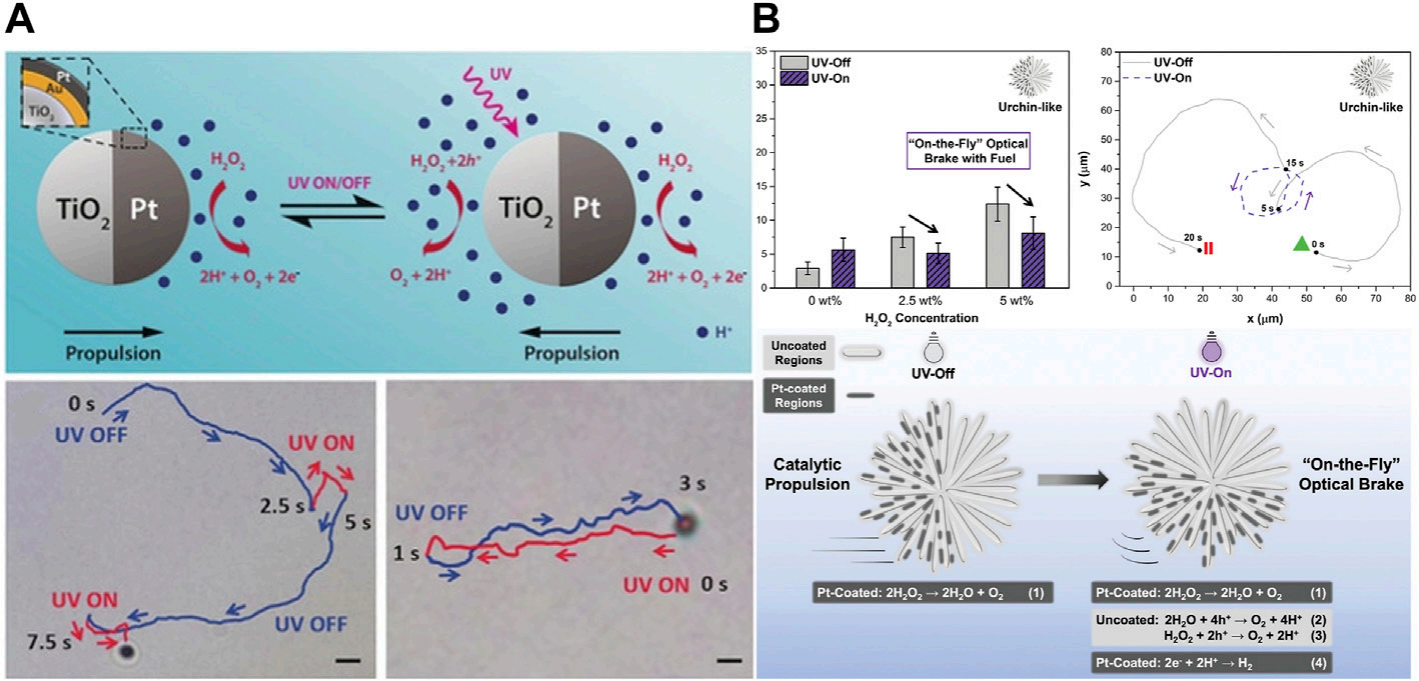
Examples of microrobots with optical brakes. **(A)** A balance of catalytic (Pt side) and optical (TiO_2_ side) reactions to halt the motion. Reproduced with permission (Chen et al., 2018b) Copyright 2018 Wiley-VCH. **(B)** Motion and on-the-fly optical brakes are achieved by shape engineering of urchin-like TiO_2_ microrobots. Reproduced with permission (Oral et al., 2022). Copyright 2022 Wiley-VCH.

The ability to accelerate or decelerate microrobots while performing their tasks is very important for various applications. To achieve this, a tubular micromotor made of CdS quantum dots/C_60_ was fabricated with a catalyst (Pt, Pd, or MnO_2_) embedded in it (María Hormigos et al., 2019). The quantum dots made this micromotor responsive to a broad spectrum of visible light. The combination of opto-catalytic propulsion enabled these researchers to “on-the-fly” accelerate these micromotors by shining light on the chemically propelled microrobots. Specifically, the micromotor with Pt as a catalyst moved at a speed of 670 ± 61 μm/s in a 5% aqueous solution of peroxide. The speed was increased to 950 ± 70 μm/s upon UV light (385 nm) illumination, 1,058 ± 72 μm/s under blue light (470 nm), and 870 ± 60 μm/s when exposed to green light (550 nm). The micromotors having Pd or MnO_2_ as their catalytic engines also showed similar speed enhancement trends. However, these microrobots lack the function of decreasing speed. To realize this feature, Chen and colleagues introduced the concept of a built-in optical brake for chemically powered micromotors (Chen et al., 2018b). They designed a TiO_2_/Au/Pt Janus micromotor that could be propelled optically or chemically. The micromotors showed propulsion in opposite directions depending on the Pt-catalyzed H_2_O_2_ decomposition on one side or the UV-activated TiO_2_ propulsion on the other side of the Janus micromotor (Figure 7A). A simultaneous actuation by both modes resulted in a competition for directionality and offered a control over direction by varying the experimental conditions. The micromotors were completely stopped when the two opposing forces were in equilibrium. This balance of opposing forces for “on-the-fly” optical brakes has also been utilized by Oral and co-workers (Oral et al., 2022). The researchers on this project fabricated urchin-like TiO_2_ microparticles and half coated them with Pt. The microrobots were propelled chemically in the absence of light; however, on light illumination (365 nm, 330 mW/cm^2^), the microrobots stopped due to an optical braking effect of the light-triggered chemical reactions on the uncoated TiO_2_ (Figure 7B). The authors also investigated the role of surface roughness and found that, unlike those with rough surfaces, Pt/TiO_2_ microrobots with smooth surfaces got accelerated with the application of light. Specifically, the micromotors moved at a speed of 6.3 ± 1.3 μm/s under fuel free conditions upon UV light exposure. In a 2.5% H_2_O_2_ solution without UV light, these particles showed a propulsion speed of 7.0 ± 2.9 μm/s while this speed was increased to 17.4 ± 4.3 μm/s when UV light was turned on. At 5% H_2_O_2_, the speed was further increased to 29.0 ± 3.4 μm/s. Similarly, researchers working in Hest’s group have recently reported supramolecular nanomotors operated by a light-triggered thermophoresis and enzyme-catalyzed H_2_O_2_ decomposition (Shao et al., 2022). They demonstrated a “seesaw effect” motion and a complete halting of the moving colloids by counterbalancing the opposite propulsion forces.

Another intriguing type of micromotors based on multiengine was fabricated by Yuan and co-workers (Yuan et al., 2020). The micromotors were manufactured by wrapping gold-sputtered polymeric particles with 2D nanomaterials. Then different engines were assembled onto these micromotors. Specifically, the researchers employed Pt or MnO_2_ nanoparticles as catalytic engines, Fe_3_O_4_ nanoparticles as the magnetic engines, and CdSe@ZnS quantum dots as the light engines. They also studied the combinations of different actuation modes, i.e., magneto-catalytic, opto-catalytic, and opto-magneto-catalytic modes. The combination of these modes allowed them to demonstrate an on-demand accelerating and braking system. They concluded that a combination of bubble-light-magnetic modes is effective in controlling the braking or acceleration by choosing a specific wavelength of the incident light (385, 470, or 550 nm) and catalyst type (Pt or MnO_2_).

Magnesium-based water-driven micromotors are a special class of light-actuated fuel-free microbots. They consume water as a fuel and typically move by H_2_ bubble recoil (Chen et al., 2018a). In one of the earliest examples of Mg-based micromotors, Li et al. (Li et al., 2014) showed that TiO_2_/Au/Mg microspheres could photocatalytically degrade biological and chemical warfare agents.

#### 2.2.2 Opto-acoustic

The combination of light and acoustic actuation methods has also been studied for microrobots. Zhou and co-workers utilized an opto-acoustic approach to control the aggregation and separation of light-responsive micromotors (Zhou et al., 2018). They achieved the aggregation of their active colloids at pressure nodes of the acoustic field. Moreover, the illumination of light on these aggregates resulted in their separation and dispersed the micromotors in a collective “firework” behavior. Similarly, researchers lead by Wang have reported a structure dependent collective behavior of opto-acoustic microrobots (Tang et al., 2019). They fabricated bowl-shaped Au/TiO_2_ micromotors and controlled their motion by changing the inner and outer positions of Au and TiO_2_. The microbowls could be propelled either by acoustic force or light-induced self-phoretic motion. An internal Au surface resulted in the same direction of motion for both the acoustic and optical actuations, however, an internal TiO_2_ surface resulted in a competition between the two actuation modes. Specifically, the micromotors moved with a speed of 11.35 μm/s in an acoustic field of 2.66 MHz intensity. Upon UV irradiation (365 nm), the micromotors slowed down to 5.1 μm/s at light intensity of 0.4 W/cm^2^. A further increase in the light intensity to 0.8 W/cm^2^ reversed the direction of motion and particles moved with a speed of 21.8 μm/s. Additionally, the light modulations also allowed expansion and compaction of different ensembles of the microrobots.

#### 2.2.3 Acousto-catalytic

Wang and co-workers demonstrated the directional control and collective behavior of Au-Ru microrods by a combination of catalytic and acoustic actuations (Wang et al., 2015b). The direction of these rods was controlled by switching between the fields i.e., the rods moved with Au end leading when they were powered chemically by H_2_O_2_, while Ru end lead the motion in an acoustic field. Moreover, the researchers were able to control the reversible aggregation of these microrods by varying the magnitude of acoustic power. Similarly, a team lead by Wang (Xu et al., 2015) demonstrated the controlled swarm movement and separation of Au-Pt nanowire motors by changing the frequency of the acoustic field. This effect relied on the interaction between the individual nanomotors, and the pressure gradients generated by the acoustic force, which triggers rapid migration and assembly around the nearest pressure node.

As mentioned earlier, rheotactic motion is very important for the application of microrobots. The acousto-catalytic combination employed by Mallouk’s team (Ren et al., 2017) was demonstrated to be fruitful to achieve both the positive and negative rheotaxis. The researchers fabricated an acoustofluidic device in which bimetallic microrobots were propelled chemically or acoustically. It was shown that combining the orientational effect of chemical power and directional effect of acoustic power could result in both the positive and negative rheotaxis.

#### 2.2.4 Electro-catalytic

Magneto-catalytic propulsion has been a popular hybrid system; however, it requires a magnetic segment in the microrobots and bulky magnetic setups for the actuation. As an alternative, researchers in Fan’s group investigated the application of an electric field to guide the motion of catalytically-driven microrobots (Guo et al., 2018). The researchers showed a precisely controlled manipulation of chemically powered nanomotors by applying an electric field in 3D. They utilized DC electric field to control the speed of microrobots and an AC electric field to guide the alignment of Pt-Au microrods. These microrobots were employed for cargo delivery and powering nanomechanical devices for their continuous rotation. A recent interesting example of a multistimuli-responsive microrobot was reported from Yossifon’s group (Das et al., 2022). These researchers created a triple-hybrid micromotor powered by catalytic, magnetic, and electric actuations. They synthesized Mg-based micromotors containing different layers of other constituents i.e. Ti/Ni/Ti/TiO_2_/Mg. The micromotors were rapidly switched from chemical to electrical propulsion by using magnetic rolling between two chambers of a microfluidic device. The authors also showed the trapping and delivery of biological cells by these microrobots which make them good candidates for future biomedical applications.

## 3 Conclusion

We have reviewed the most recent developments in the multistimuli-responsive microrobots. It is evident from this literature survey that the field of microrobotics is making a rapid transition from the fundamental research towards its promised applications. To realize this, a combination of different actuation methods has been very useful. The scale of future potential applications of microrobots suggests that it is only a beginning and more future effort will be devoted to the multistimuli-enabled microrobots. We speculate that the microrobotic research community will continue to work on biomedical and environmental applications, however, many uncharted territories will also be explored as the field continues to grow. More focus will be devoted to address the current challenges such as biocompatibility, microrobot recovery after performing their tasks, and achieving a better control over swarm motion in both the vertical and horizontal directions.
